# Singular Death from Chloroform at Yardley

**Published:** 1897-11

**Authors:** 


ARTICLE V.

### SINGULAR DEATH FROM CHLOROFORM AT YARDLEY.

Mr. A. H. Hebbert held an inquest at the Yardley Arms Hotel, on the body of Norris Wilmott Kenyon, aged 17, who lived with his mother, a widow, at Ashton Hill Cottage, Yardley, and who died from chloroform he had taken to alleviate the pain of toothache, to which he was a victim.

Annie Kenyon, 45 Edgware Road, London, said the deceased was her brother, her husband taking her name for the purpose of their business. Her brother was the last who saw him at eight o'clock on Thursday night. He looked very well, having just had a holiday, from which he returned on Wednesday. He suffered from neuralgia, which came on after he had gone to bed. She had found chloroform in his drawer, and asked him if he kept it about in those quantities. He said, "Yes." She told him that it was very careless, and that people who used chloroform sometimes had accidents. He replied that it was all right. He knew all about it, and used it for toothache. Chloroform was sometimes used in the factory, and her brother would have occasion to go to Southall's—from whom the chloroform was obtained—to order acids, etc., for the business. She had no reason to think that he would commit suicide. She spoke to her mother about his having chloroform, and the mother told her that when she had taken some away, he replied that it was no use doing that, as he could get quarts if he wanted. He would always have his own way. He used to suffer from neuralgia after a long ride. Another brother of hers, aged about twenty-four, shot himself about ten years ago, about ten miles from Brighton.

Mrs. Pepperell, housekeeper at the house of the deceased's mother said deceased had for long been in the habit of taking chloroform for neuralgia.

Herbert Hollich Kenyon, managing director of a funeral company, stated that he was staying at Ashton Hill Cottage. He went to deceased's bed-room a little after eight o'clock. His brother-in law was lying in bed in quite a natural position. Witness saw by the appearance of deceased's face that something serious had happened. He felt his heart, and found he was quite dead, although the body was just warm. A handkerchief was over the nose and mouth. Deceased had told him that chloroform was a pleasant thing to take. He drank it in small quantities, and witness had warned him as to the danger. There was no reason why the deceased should commit suicide. Witness and deceased chloroformed a litter of kittens a fortnight ago.

The evidence of Dr. Pugh showed that death was due to the inhalation of chloroform, although it was quite possible that the actual cause of death was suffocation by vomited matter in the mouth rather than poisoning by chloroform. The excessive rigidity of the body indicated that the amount of chloroform taken was not very large.

The jury unanimously returned a verdict of "Death from misadventure," and offered their deepest sympathy with the relations.—*British Journal of Dental Science.*

